# Human Blood Serum Inhibits Ductal Carcinoma Cells BT474 Growth and Modulates Effect of HER2 Inhibition

**DOI:** 10.3390/biomedicines10081914

**Published:** 2022-08-08

**Authors:** Dmitrii Kamashev, Nina Shaban, Maria Suntsova, Mikhail Raevskiy, Victor Efimov, Aleksey Moisseev, Maxim Sorokin, Anton Buzdin

**Affiliations:** 1 Shemyakin-Ovchinnikov Institute of Bioorganic Chemistry, 117997 Moscow, Russia; 2 I.M. Sechenov First Moscow State Medical University, 119991 Moscow, Russia; 3 Moscow Institute of Physics and Technology, Dolgoprudny, 141701 Moscow, Russia; 4 Oncobox Ltd., 121205 Moscow, Russia; 5 OmicsWay Corp., 340 S Lemon Ave, 6040, Walnut, CA 91789, USA

**Keywords:** trastuzumab, targeted therapy, HER2 overexpression, ERBB2 amplification, BT474, breast cancer, ductal carcinoma, human serum endogenous factors, personalized medicine, RNA sequencing, molecular pathway analysis

## Abstract

Trastuzumab, a HER2-targeted antibody, is widely used for targeted therapy of HER2-positive breast cancer (BC) patients; yet, not all of them respond to this treatment. We investigated here whether trastuzumab activity on the growth of HER2-overexpressing BT474 cells may interfere with human peripheral blood endogenous factors. Among 33 individual BC patient blood samples supplemented to the media, BT474 sensitivity to trastuzumab varied up to 14 times. In the absence of trastuzumab, human peripheral blood serum samples could inhibit growth of BT474, and this effect varied ~10 times for 50 individual samples. In turn, the epidermal growth factor (EGF) suppressed the trastuzumab effect on BT474 cell growth. Trastuzumab treatment increased the proportion of BT474 cells in the G0/G1 phases of cell cycle, while simultaneous addition of EGF decreased it, yet not to the control level. We used RNA sequencing profiling of gene expression to elucidate the molecular mechanisms involved in EGF- and human-sera-mediated attenuation of the trastuzumab effect on BT474 cell growth. Bioinformatic analysis of the molecular profiles suggested that trastuzumab acts similarly to the inhibition of PI3K/Akt/mTOR signaling axis, and the mechanism of EGF suppression of trastuzumab activity may be associated with parallel activation of PKC and transcriptional factors ETV1-ETV5.

## 1. Introduction

HER receptor tyrosine kinase family includes four members: EGFR (HER1), HER2 (neu), HER3, and HER4 proteins [1,2,3,4]. The HER receptors are essential for mediation of proliferative signals to cells [5,6,7,8]. Several growth factors are known to be able to bind these receptors and activate them, called HER ligands [9,10,11,12]. Overexpression of the HER receptors as well as their hyperactivating mutations are essential for abnormal proliferative signaling in a variety of cancers [13,14,15,16,17,18].

HER2-overexpressing breast cancer (HER2+ BC) represents ~15–20% of all breast cancers [19,20]. For therapy of HER2+ BC, humanized monoclonal antibody trastuzumab has been approved as the standard of care [21,22,23,24,25,26,27]. In addition, HER2-targeting antibodies were also effective as the second line of therapy for 7–15% of patients with advanced ovarian cancers [28] and with gastric cancer [29]. However, not all patients with tumors expressing high levels of HER2 respond to trastuzumab [30,31,32,33], and many cancers develop resistance to such treatment [34,35].

In cell culture studies, the rescuing effects of HER ligands (epidermal growth factor (EGF), neuregulin (NRG)) on cells treated with HER-targeting drugs were demonstrated [36,37,38,39]. Endogenous EGF and other HER ligands are present in cancers and can rescue cells during HER-targeted therapy. Thus, tumor response can be affected by a variety of extracellular factors present in the patient’s body, particularly in human peripheral blood. These factors substantially influence the anti-tumor action of the drugs during treatment [36,37,38,39].

Here, we investigated the impact of human serum on the anti-proliferative activities of trastuzumab on the in vitro cultured HER2+ cells. BT474 cells, which are HER2+ BC cells overexpressing HER2 [40], have been utilized extensively as a cell culture model to study HER2+ BC response to trastuzumab treatment [7,41]. We measured BT474 colony formation ability (clonogenicity) in the presence of human peripheral blood sera taken from fifty human donors and studied its influence on the activity of trastuzumab and its donor-to-donor variation. We also investigated the modulation of clonogenicity by EGF, a major EGFR ligand present in human blood. We showed that EGF diminishes the effect of trastuzumab in a dose-independent manner even at 1 ng/mL, which corresponds to its physiological concentration in the human blood [42,43]. In contrast, we demonstrated that human blood sera in combination with trastuzumab ihibit BT474 cell growth in synergistic, antagonistic or additive manner.

To understand the possible molecular mechanisms of trastuzumab cell growth inhibition and resistance, extensive studies on gene expression profiling have been performed previously using HER2-overexpressing BT474 and SKBR3 cells [44,45,46]. The comparison of gene expression of HER2+ BC tumors that respond or do not respond to trastuzumab treatment revealed molecular pathways important for successful tumor therapy [35,47]. Thus, we performed RNA sequencing for HER2+ BT474 cells treated with human blood serum, trastuzumab, EGF or EGF and trastuzumab combination. Bioinformatic analysis of the molecular profiles suggested that trastuzumab acts similarly to the inhibition of PI3K/Akt/mTOR signaling axis, and the mechanism of EGF suppression of trastuzumab activity may be associated with parallel activation of PKC and transcriptional factors ETV1-ETV5.

## 2. Materials and Methods

### 2.1. Cell Culture

The ductal carcinoma cell line BT-474 (ATCC HTB-20) cells were cultured at 37 °C and 5% CO_2_ in RPMI-1640 (Paneco, Russia) supplemented with 10–15% FBS (Gibco BRL) and 2 mM L-glutamin, 4.5 g/L glucose, 1% penicillin-streptomycin and 10 ng/mL insulin.

### 2.2. Clonogenicity Assay (Colony Formation Assay)

Cells were plated in 25 cm^2^ flasks, 10,000 cells per flask. Alternatively, the cells were plated on six-well culture plates 2000 cells per well. The plates with equal number of cells seeded were incubated for 24 h before treatment with EGF or anti-HER2 drugs, or human sera. Downstream 21-day incubation of the cells led to the formation of colonies, defined as more than 50 cells. The medium was discarded, and the cells were fixed using 4% formaldehyde for 10 min, and the cells were stained using 0.5% of crystal violet in 60% methanol and 0.5xPBS for 15 min and washed with water. The colonies were detected and counted by the openCFU software [48]. The colony formation (in %) was calculated as a ratio of the number of colonies formed by treated and untreated cells. All experiments were conducted with at least three independent replicates.

### 2.3. Cell-Cycle Analysis

The cells were plated on six-well culture plates at a density of 50,000 cells per well. Cells were incubated for 16 h, and drugs were added as indicated. Following 3–4 days of treatment, cells were detached with trypsin. Cells were washed with PBS (by centrifugation at 100× *g*) twice. The cell pellet was fixed with ethanol (70%) at –20 °C for 30 min. The cells were centrifuged for 10 min at 3800 rpm (1000× *g*); the pellet was washed with PBS and resuspended in 0.2 mL PBS containing 10 μg/mL of RNase A II and 2 μg/mL of propidium iodide. Cells were incubated at room temperature for 30 min, protected from light. The samples were analyzed by flow cytometry (FACS BD Accuri C6 Plus).

### 2.4. Cell Treatment for RNA Sequencing

Cell suspension was diluted to 27,000 cells per ml. The cells, 5 mL of cell suspension, were plated on 25 cm^2^ culture flasks. From the same cell suspension, 0.5 mL of cells per well were plated on wells of 6-well culture plates for the growth rate measurement. Cells were incubated for 16 h, and drugs were added as indicated. Examples: trastuzumab, 160 µL of the freshly diluted solution in growth media per flask to a final concentration of 3.6 µg/mL; EGF, 100 µL of freshly diluted solution in growth media per flask to a final concentration of 8 ng/mL; 300 µL human blood serum per flask to a final concentration of 5.5%. To the control wells, 1/10 volume of the same drug solutions was added. Cells were treated with drugs in the flasks for 48 h before harvesting samples for RNA seq and for 10 days for cell counting. For RNA seq, the cells were detached with trypsin, washed with PBS, and cell pellets were stored at −70 °C in RNAlater solution before analysis. The human serum used for RNA seq analysis was a pool of eight healthy donors (all women), sd: 2, 14, 15, 17, 18, 19, 20, 21.

### 2.5. HER-Targeted Drugs, EGF and Human Serum Samples

Trastuzumab (Herceptin), dry powder, was purchased from Roche, stored at 4 °C; rhEGF, dry powder, was purchased from SCI store (Moscow, Russia) and stored at −20 °C. Human serum samples were obtained from the blood samples (16 mL each) obtained from fifteen independent healthy 23–64 y.o. female donors and twenty 31–69 y.o. female donors with HER2-positive breast cancer diagnosed at the Blokhin National Medical Research Center of Oncology of the Ministry of Health of Russia, Moscow. In each case, donor blood was collected in two 8 mL Vacuette tubes containing the pro-coagulant and gel (Greiner). Serum was prepared 4–24 h after blood collection; the tubes were centrifuged at 2500 rpm for 15 min, and the sera were aliquoted and stored at −75 °C. For all human biomaterials, written informed consent to participate in this study and to communicate the results in this report was collected from the corresponding donors. The consent procedure and the design of the study were approved by the ethical committee of the Vitamed Clinical Center, Moscow.

### 2.6. Preparation of Libraries and RNA Sequencing

RNA libraries were generated and sequenced according to Suntsova et al. [49]. RNA was extracted using RecoverAll™ Total Nucleic Acid Isolation Kit (Invitrogen). RNA concentrations were measured with Qubit RNA Assay Kit, and Agilent 2100 bioanalyzer was used to measure RNA Integrity Number (RIN). Depletion of ribosomal RNA was performed using RNA Hyper Kit (Roche), and then, library concentrations and fragment length distributions were measured with Qubit (Life Technologies) and Agilent Tapestation (Agilent), respectively. The RNA seq was performed using Illumina NextSeq 550 engine for 50 bp single-end reads and approximately 30 million raw reads per sample using standard protocol.

### 2.7. RNA Sequencing Data Processing

Gene expression profiles were initially processed according to Suntsova et al. [49]. Differential expression analysis was performed using DESeq2 [50]. Genes that were considered significantly differentially expressed had to pass a threshold of FDR-adjusted *p*-values < 0.1. GO enrichment analysis was conducted using R packages clusterProfile (v.4.2.1) and org.Hs.eg.db (v.3.8.2). PCA and visualization were performed for log10 transformed counts of all genes using pca2d R (v.3.6.0) and prcomp software. Connectivity map (CMAP) analysis was performed as described by Subramanian et al. [51]. Gene-regulatory network analysis was performed as described by S. Jung et al. [52]. Pathway activation levels (PALs) were calculated and visualized with the Oncobox bioinformatical platform [53]. The molecular function of pathway components was algorithmically annotated according to Ref [54].

## 3. Results

### 3.1. Human Blood Serum Samples Affect Colony Formation of BT474 Cells in a Donor-Specific Manner

In this study, we measured the impacts of individual human peripheral blood serum samples on the cell colony formation of HER2-overexpressing ductal carcinoma BT474 cells. The influence of the blood sera from both healthy individuals and HER2+ BC patients on the cell growth was studied alone or in the presence of trastuzumab.

The peripheral blood serum specimens were obtained from healthy donors and individuals with diagnosed HER2+ BC (Table S1). For all the samples tested, the growth media contained heat-inactivated fetal bovine serum (FBS). For the control test, we took growth medium supplemented with 12.5% FBS, and for the tests with human sera, we used growth medium supplemented with 10% FBS and with 2.5% of the human serum. Thus, to implement this study design, we first investigated how BT474 clonogenicity depends on the FBS concentration in the growth medium. We showed that clonogenicity was changing slowly and statistically non-significantly in the range of FBS concentrations between 7.5 and 17.5% (Figure S1).

We then tested BT474 clonogenicity in the growth medium with 10% FBS and 2.5% human serum (or with 2.5% of additional FBS for the controls). We used human peripheral blood serum specimens obtained from 16 healthy female (sd) and 1 male (sm) donors and from 33 HER2+ BC patients (sp), 50 human serum samples in total. Female donor healthy samples were mostly used to avoid gender-specific bias when comparing results between the BC and healthy groups.

**Figure 1:**
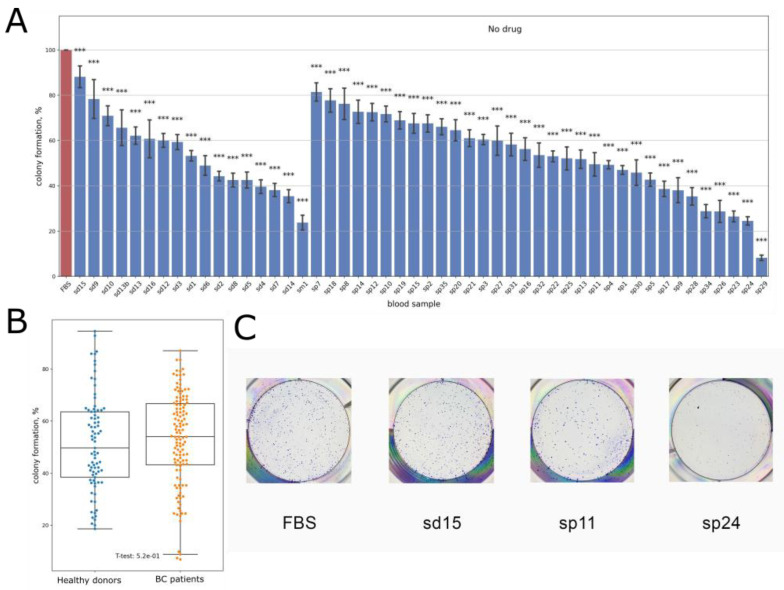
(**A**,**B**) Colony formation by BT474 in the growth media containing 10% heat-inactivated FBS supplemented with 2.5% human blood serum samples; samples sd correspond to healthy donors; samples sp correspond to HER2+ breast cancer patients. Colony formation is normalized to the growth media containing 12.5% heat-inactivated FBS without drug (column FBS). The data are presented as mean ± standard deviation from three independent replicates (see Table S2). (**C**) Representative photographs of stained clonogenicity assay results used for colony number calculations. ***—*p*-value < 0.001.

We found that for all the samples tested, the human serum had either neutral or inhibitory effects on the colony formation (Figure 1). In particular, BT474 clonogenicity was statistically significantly inhibited by all healthy (n = 17) and BC (n = 33) serum samples. Clonogenicity varied from 23 to 88% for the healthy and from 8 to 81% for the BC samples (Figure 1, Table S2), thus evidencing up to ~4-fold inhibition in healthy and up to ~10-fold inhibition in cancer samples compared to the control containing 12.5% FBS only.

Overall, the mean clonogenicity for healthy donor samples was 53.8% compared to 53.3% for the BC samples; the difference was not statistically significant. Clonogenicity was calculated as a ratio of the number of colonies formed by human-sera-treated cells and cells treated with FBS only.

### 3.2. Human Blood Serum Can Enhance and Diminish Inhibitory Effect of Trastuzumab on BT474 Clonogenicity

**Figure 2:**
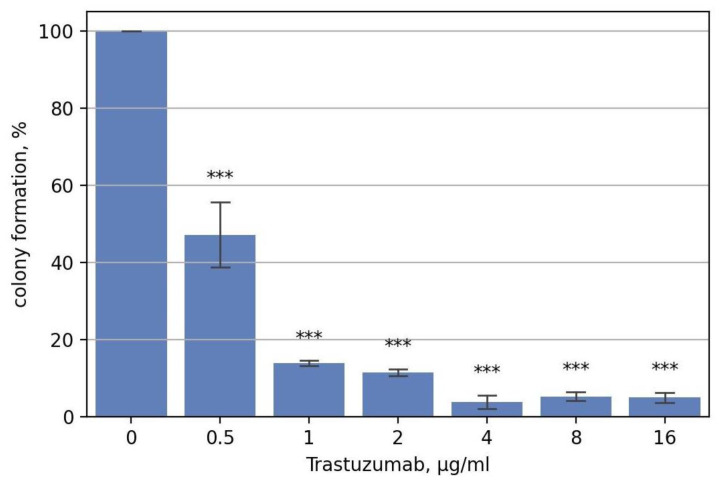
BT474 cells colony formation upon the treatment with trastuzumab (concentration is given in µg/mL) in the presence of 12.5% of heat-inactivated FBS. The data are presented as mean ± standard deviation from three independent replicates. ***—*p*-value < 0.001.

Invasive ductal carcinoma cell line BT474 is known to overexpress HER2 receptors (~2.75 × 10^6^ HER2 molecules per cell [55] vs., e.g., ~4 × 10^4^ for A431 cells) and is sensitive to specific HER2-blocking therapeutic antibody trastuzumab [40]. We estimated IC50 for trastuzumab as approximately 0.5 µg/mL, which was close to the previous estimate in the colony-forming assay (0.2 µg/mL [56]) (Figure 2). At 2 µg/mL trastuzumab, the clonogenicity apparently decreased by 10-fold compared to non-drug level.

**Figure 3:**
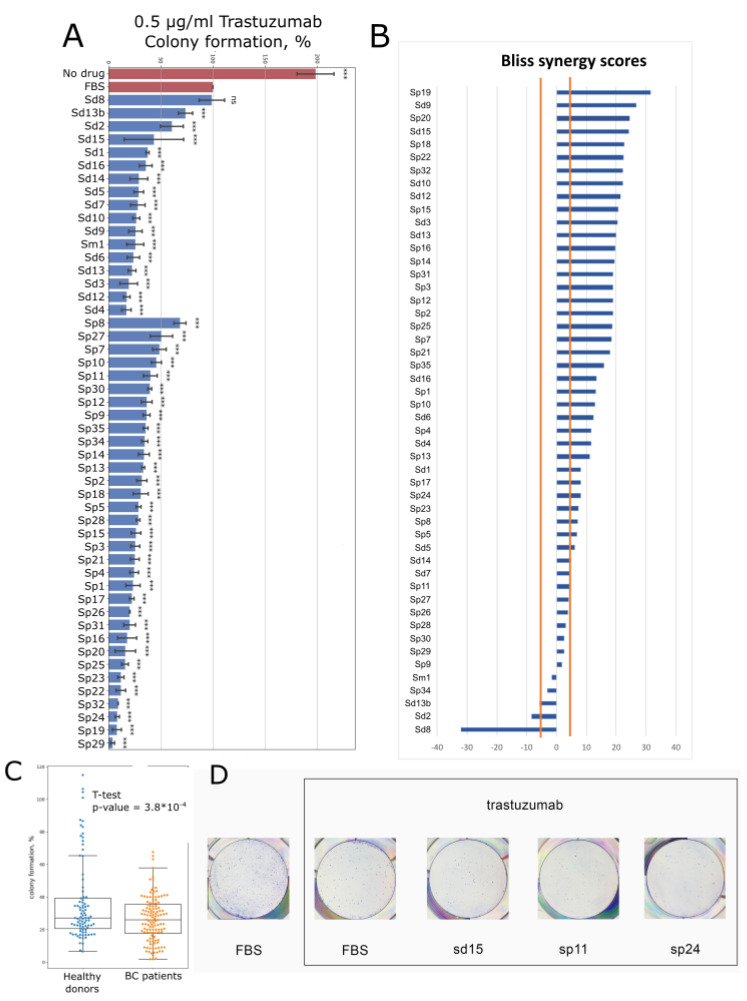
(**A**) BT474 cells colony formation upon the treatment with trastuzumab (0.5 µg/mL) in the presence of 2.5% human blood serum. Samples sd correspond to healthy donors; samples sp correspond to HER2+ BC patients. FBS shows clonogenicity in the presence of 12.5% of heat-inactivated FBS only. Colony formation is normalized to the growth media containing only 12.5% heat-inactivated FBS and trastuzumab (FBS). No drug represents clonogenicity without drug and human sera. The data are presented as mean ± standard deviation from three independent replicates (see Table S2). (**B**) Influence of human blood serum on trastuzumab inhibition of BT474 cells colony formation. Bliss synergy scores (BS) for serum and trastuzumab interaction were calculated using the data presented in Figure 1 and Figure 3A. If synergy score BS < −5, the interaction is thought antagonistic; if BS > 5, the interaction is thought synergistic; while at BS between −5 and 5, it is thought additive [57,58,59]. ***—*p*-value < 0.001; ns—non-significant. (**C**) BT474 cells colony formation upon the treatment with trastuzumab (0.5 µg/mL) in the presence of 2.5% human blood serum from healthy donors (blue) vs. BC patients (orange). (**D**) Representative photographs of stained clonogenicity assay results were used for colony number calculations.

We then measured the trastuzumab effect on colony formation in the media supplemented with 2.5% peripheral blood serum samples along with 10% FBS and in the control medium containing 12.5% FBS (Figure 3, Table S2). Trastuzumab concentration was chosen as 0.5 µg/mL, which decreased BT474 cells colony formation ability by ~51% compared to the control medium (Figure 3).

To further investigate whether the human serum supplements provide synergistic, additive or antagonistic effects to the drug, we measured the Bliss synergy score (BS) with the discriminating threshold of BS > 5 for the synergistic effect and BS < −5 for the antagonistic effect [57] (Table S2). 

We found that the inhibitory effect of trastuzumab and healthy human serum samples on cell clonogenicity was antagonistic for only three samples (lesser growth inhibition after combined treatment with trastuzumab and human serum than with trastuzumab and serum samples alone) (Table S2, Figure 3B). In total, 3 serum samples showed additive while the other 11 showed synergistic effect with trastuzumab. Finally, we found an approximately 6-fold variation in BT474 clonogenicity for the healthy donor serum samples supplemented with trastuzumab (Figure 3, Table S2).

For BC patient serum samples, the inhibitory effects with trastuzumab were synergetic for 25 samples. Eight samples showed additive effects, and no samples—antagonistic effects (Table S2, Figure 3B).

We conclude, therefore, that human serum can inhibit growth of BT474 cells antagonistically or synergistically with trastuzumab. Finally, we detected an approximately 14-fold variation in BT474 clonogenicity observed for the 33 BC patient serum samples and a 6-fold variation for 17 healthy donor serum samples when added in combination with trastuzumab (Figure 3, Table S2).

### 3.3. EGF Interferes with Trastuzumab Effects on BT474 Cell Growth

**Figure 4:**
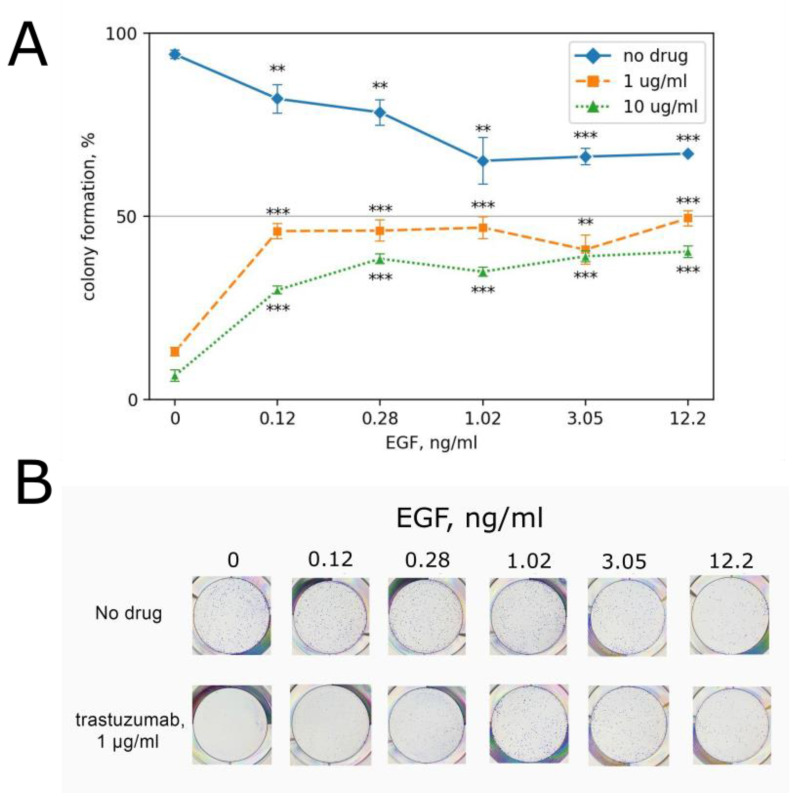
(**A**) EGF concentration dependence of BT474 clonogenicity in the absence and in the presence (1 µg/mL and 10 µg/mL) of trastuzumab. Clonogenicity is normalized in the results for the control growth medium without trastuzumab. (**B**) Representative photographs of stained clonogenicity assay results were used for colony number calculations. **—*p*-value < 0.01; ***—*p*-value < 0.001.

In our experiments, BT474 cells clonogenicity decreased to ~64% in the presence of EGF at concentrations exceeding 1 ng/mL compared to non-drug level. Clonogenicity did not significantly depend on EGF concentration between 1 and 100 ng/mL of EGF (Figure 4), in contrast to A431 cells, whose growth could be completely inhibited by EGF concentrations starting from 35 ng/mL [60]. BT474 cells are highly sensitive to trastuzumab with IC50 ~0.5 µg/mL, while a plasma level of trastuzumab is 20.2 µg/mL during long-term cancer treatment and patient repair after surgical operations [60].

Previously, we and others observed that EGF can suppress the activities of EGFR-targeted drugs in EGFR-overexpressing cells [36,37,38,39]. Thus, here, we explored whether it can inhibit trastuzumab cytotoxicity in the HER2-overexpressing cell line BT474. To this end, BT474 clonogenicity was measured in the presence of trastuzumab and EGF (Figure 4).

We found that EGF clearly restricts the inhibitory effects of trastuzumab on BT474 cell growth. For example, trastuzumab at the concentration of 1 µg/mL (without EGF) decreased colony formation to ~12% of the control non-drug clonogenicity, and the addition of EGF increased the number of colonies by ~4-fold, to ~46% of the control level. At a ten-fold greater concentration of trastuzumab (10 µg/mL), leading to only ~7% clonogenicity without EGF, the addition of EGF augmented clonogenicity by ~5-fold, to 35% of non-drug level (Figure 4).

Interestingly, the observed EGF ability to prevent trastuzumab growth inhibition was relatively dose independent; in the range of EGF concentrations of 0.1–12 ng/mL, approximately the same BT474 clonogenicity was detected. Actual EGF concentration in human blood varies among individuals in the range of 0.3–1.7 ng/ml [42,43]. Thus, our data evidence that the human blood EGF can have protective effects on cancer cells that counteract with trastuzumab cytotoxicity.

### 3.4. BT474 Cell-Cycle Progression Alterations Caused by Trastuzumab, EGF and Human Sera

The anti-HER2 drugs are known to inhibit cell-cycle progression by causing the G1 phase arrest [61]. In this study, the treated and control BT474 cells underwent fluorescence-activated cell sorting (FACS) analysis. In 10 ng/mL trastuzumab treated cells, we observed a G0/G1 phase shift to 72.8% from the baseline of 66.9% (Table 1). This treatment inhibited cell growth to only 7% of the baseline level in the clonogenicity tests. When trastuzumab was added along with EGF, clonogenicity increased to 28% of the non-drug level (Table 1). We found that the proportion of cells in the G0/G1 fraction did not shift back to non-drug level and remained shifted up from 66.9 to 71.0%. The proportion of cells in the DNA synthesis (S) phase did not change significantly in the presence of EGF (9.5% vs. 9.9%) and was decreased by trastuzumab treatment (to 6.6%) and simultaneous trastuzumab + EGF treatment (to 7.1%).

In the media supplemented with human sera, BT474 cell growth was inhibited (Figure 1). Thus, we measured cell-cycle distribution of the cells in the media supplemented with sera from seven healthy donors (Table 1). We found that for all seven tested samples, the proportion of cells in the G0/G1 phase decreased (by 2.0–6.7%) with the mean effect of −4.44%, while the proportion of cells in the G2/M phase instead increased (by 0.8–4.7%) with the mean value of 3.14%. Increased cell content in the G2/M phase can be responsible for the human serum inhibitory effect [62].

### 3.5. Variation of HER Ligand Concentrations between Experimental Human Blood Samples

**Figure 5:**
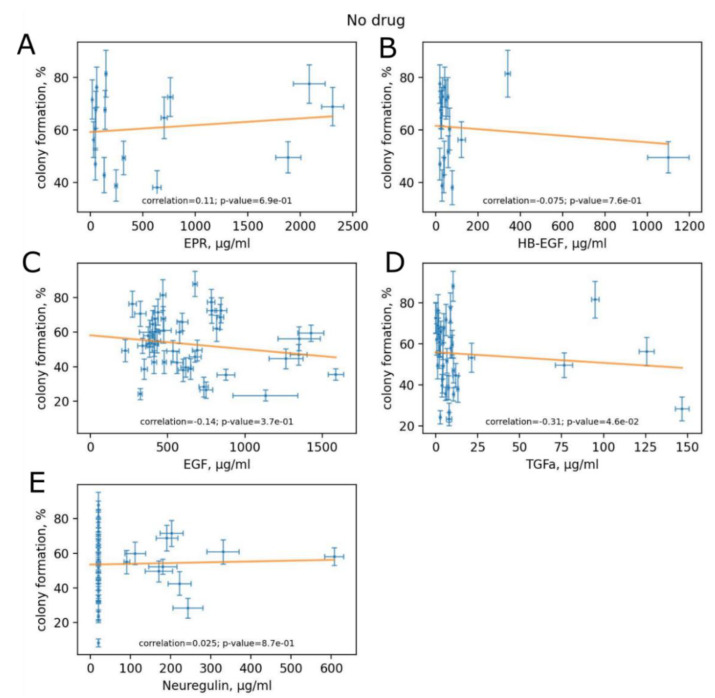
Concentrations of HER ligands in serum vs. clonogenicity without trastuzumab: (**A**) epiregulin; (**B**) HB-EGF; (**C**) EGF; (**D**) TGFa; (**E**) neuregulin. The data are presented as mean ± standard deviation from three independent replicates.

**Figure 6:**
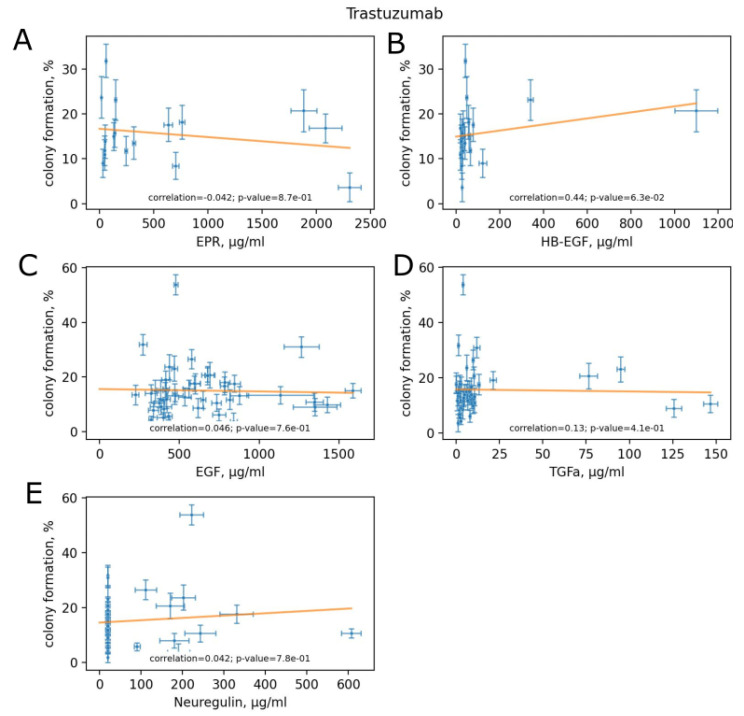
Concentrations of HER ligands in serum vs. clonogenicity with trastuzumab: (**A**) epiregulin; (**B**) HB-EGF; (**C**) EGF; (**D**) TGFa; (**E**) neuregulin. The data are presented as mean ± standard deviation from three independent replicates.

We showed increased clonogenicity of BT474 cells exposed to trastuzumab in the presence of recombinant human EGF compared to trastuzumab only. Thus, we measured, with the ELISA method, the human blood concentrations of five major HER ligands: EGF, transforming growth factor alpha (TGFa), neuregulin (NRG), heparin-binding EGF-like growth factor (HB-EGF) and epiregulin in the samples of healthy donors and HER2+ BC patients used in clonogenicity assays. We then assessed whether the clonogenicity was associated with the serum concentrations of HER ligands with and without trastuzumab (Figure 5 and Figure 6). We found statistically significant, yet relatively low (−0.31), negative correlation of TGFa concentration and clonogenicity in the absence of trastuzumab (Figure 5D) but not when trastuzumab was added to the media (Figure 6D). No other HER ligands concentration was associated with clonogenicity, neither in the absence nor in the presence of trastuzumab (Figure 5 and Figure 6).

### 3.6. mRNA Profiling of the Cells Exposed to Human Blood Serum, EGF and Trastuzumab

**Figure 7:**
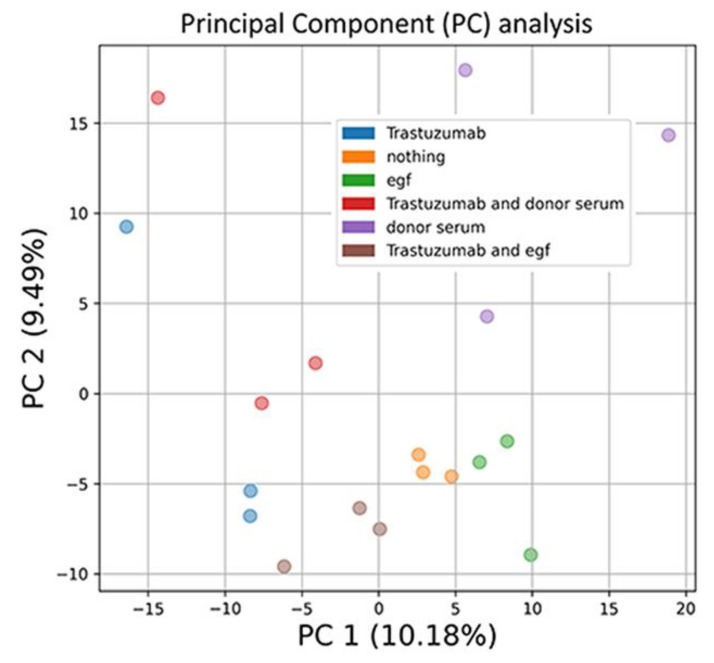
Principal component (PC) analysis of log-transformed gene expression profiles. The color key indicates functional groups of samples. The proportion of standard deviation for the respective PC is shown in brackets.

We found that trastuzumab inhibited the proliferation of HER2-positive BT474 cells, while EGF decreased the trastuzumab effect. To study the molecular mechanisms involved in EGF and human sera attenuation of the trastuzumab inhibition of BT474 proliferation, we sought to measure the gene expression level. We chose cell treatment condition where trastuzumab (4 µg/mL) arrested cell growth to about 3% compared with the control non-drug level (both with or without human sera), and EGF (8 ng/mL) and trastuzumab inhibited growth to 33%. We used six experimental conditions: (1) control (nothing), (2) EGF, (3) Trastuzumab, (4) human serum, (5) human serum plus trastuzumab and (6) EGF plus trastuzumab. The human serum used for RNA sequencing profiling was a pool of eight healthy donors (all women), sd: 2, 14, 15, 17, 18, 19, 20, 21. Cells were harvested in 72 h, mRNA was extracted, and then, cDNAs were synthetized and sequenced. All experiments were performed in triplicate. Principal component analysis of log-transformed gene expression profiles did not reveal visible batch effects (Figure 7).

**Figure 8:**
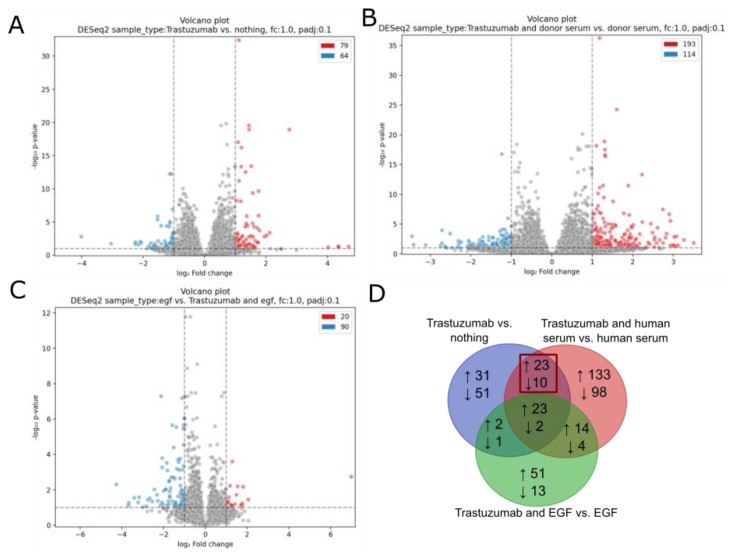
Differentially expressed genes in the comparisons (**A**) Trastuzumab vs. FBS (nothing); (**B**) Trastuzumab and donor serum vs. donor serum; and (**C**) EGF vs. Trastuzumab and EGF. Upregulated genes are shown in red (log2FC > 1, adjusted *p*-value < 0.1), downregulated genes are shown in blue (log2FC < −1, adjusted *p*-value < 0.1). (**D**) Intersection of the differentially expressed genes for the three comparisons above.

To study how EGF and human serum influenced the trastuzumab inhibitory effect, we treated BT474 cells with EGF + FBS, human serum + FBS and FBS only as a control; for these conditions, we observed similar growth rates (85%, 78% and 100%, respectively). Additionally, at the same three conditions, the gene expression was profiled for the cells simultaneously treated with trastuzumab (growth rates were 33%, 3.5% and 2.6% of drug-free level, respectively). The fold change of genes whose expression was significantly (FDR-adjusted *p*-value < 0.1; fold change > 2) increased/repressed (differentially expressed (DE) genes) by trastuzumab treatment is presented in Supplementary Table S3. We found 143 DE genes without human serum added (Figure 8A) and 307 DE genes with the addition of human serum (Figure 8B).

The DE genes identified in all the above comparisons are listed in Supplementary Table S3. Among them, 23 genes were upregulated, and 2 genes were downregulated by trastuzumab treatment in all three comparisons under study (Figure 8D).

The addition of EGF was shown to partially restore clonogenicity of BT474 cells upon trastuzumab treatment. Thus, we focused on the genes, which were altered by trastuzumab in the presence of FBS (control) and human serum but not in the presence of EGF, totaling 23 upregulated and 10 downregulated genes (marked by frame in Figure 8D). We hypothesized that these genes may be important for EGF-mediated rescue of BT474 cells from trastuzumab growth inhibition.

**Figure 9:**
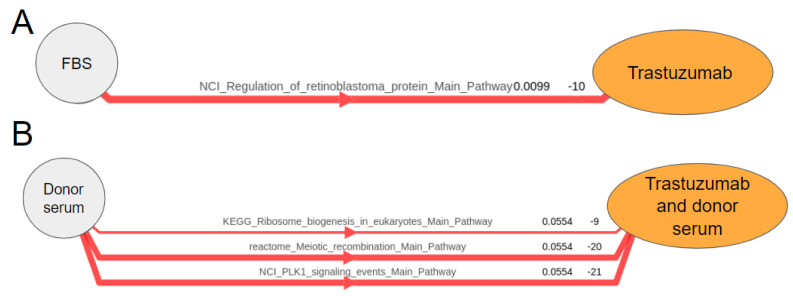
Pathway activation charts for the comparisons of BT474 cells with and without trastuzumab added. Red lines show most strongly inhibited pathways (ordered bottom to top). Thickness is proportionate to absolute value of PAL. RNA sequencing gene expression profiles of BT474 cells with and without trastuzumab were compared in the presence of (**A**) FBS only or (**B**) donor serum. PAL values and FDR-adjusted t-test *p*-values are shown to the right of the pathway names.

We also calculated the pathway activation levels (PAL) using the Oncobox software for 3044 molecular pathways and assessed statistically significantly differentially regulated pathways for the same three comparisons with and without trastuzumab. No upregulated pathways were found. The following three pathways were found to be downregulated by trastuzumab in the presence of donor serum: “Ribosome biogenesis”, “Meiotic recombination” and “PLK1 signaling events”. Only these pathways passed the pre-specified FDR-corrected *p*-value threshold of 0.1. The activation levels of other pathways were not significantly different according to this criterion. In the presence of FBS only, only one pathway was downregulated by trastuzumab: “Regulation of Rb protein pathway”. Finally, no significant pathways were found for the comparison of EGF vs. EGF and Trastuzumab (Figure 9). The latter finding is in line with the ability of EGF to rescue cells from growth inhibition by trastuzumab and, consequently, to diminish transcriptional changes. We also detected the minimal number of differentially expressed genes for this comparison (Figure 8).

**Figure 10:**
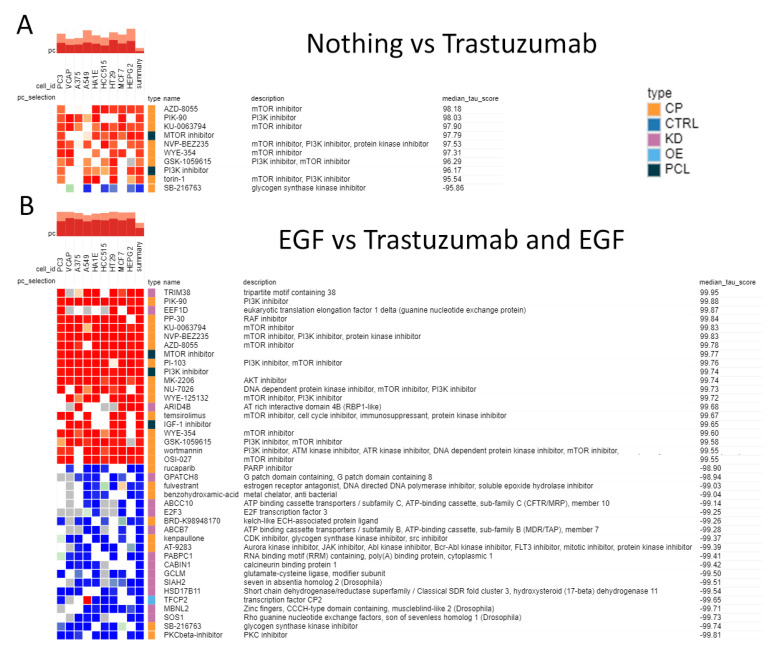
Heatmap for CMAP analysis of differentially expressed genes in the three comparisons of BT474 cells with/without trastuzumab added. (**A**) Nothing vs. Trastuzumab and (**B**) EGF vs. EGF and Trastuzumab. CP—compound, OE—overexpression, KD—knock-down, PCL—perturbagen class, CTRL—control. Red color indicates that Trastuzumab acts in a similar manner to the perturbation shown, blue color—in an opposite way. Adapted from clue.io output.

We further studied which perturbagens (compounds or genetic alterations) may act in a similar way as trastuzumab in the presence of FBS, EGF and donor serum. To this end, we used the connectivity map (CMAP) tool to analyze groups of differential genes identified in either comparison. Perturbagens with a median tau score >95 or <−95 were considered significant. We did not find any significant perturbagens in the presence of donor serum. However, in the presence of EGF or FBS only, the closest class of compounds appeared to be mTOR and PI3K inhibitors (Figure 10). At the same time, the effect of GSK3 inhibitor was the opposite to trastuzumab treatment in both cases above (Figure 10). In the presence of EGF, the most significant opposite effect to trastuzumab was obtained for the PKC inhibitor (Figure 10B). The latter was not observed for trastuzumab in the presence of FBS only, and thus, this difference may potentially be associated with the different mode of action of trastuzumab with and without EGF.

**Figure 11:**
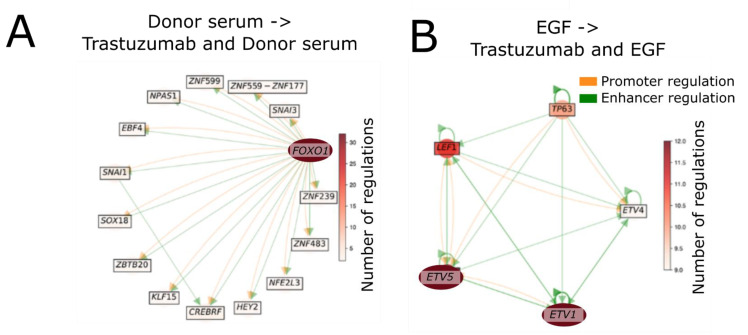
Gene-regulatory network analysis for (**A**) Donor serum to Trastuzumab and donor serum condition; (**B**) EGF to EGF and Trastuzumab condition. Promoter regulation is shown in orange, enhancer regulation—in green. Node color depth indicates the number of regulatory interactions (see color scale).

To further investigate the difference between trastuzumab treatment of BT474 cells with and without EGF, we performed gene-regulatory network (GRN) analysis according to S. Jung et al. [52]. This analysis allows detecting the network of transcription factors, which are thought to be most important for transition of cells from one state to another according to shifts in their expression profiles. We thus used this method to find the GRNs responsible for transcriptomic changes induced by trastuzumab in the presence of FBS (nothing), EGF and donor serum. The method did not reveal any transcriptional factors involved in the transition from FBS to FBS and trastuzumab-treated cells. Interestingly, the GRNs for trastuzumab were drastically different between the EGF and donor serum (Figure 11) and did not share common transcription factors. The transcription factors with the largest number of interactions were FOXO1 for donor serum and ETV1-ETV5 for EGF.

## 4. Discussion

Trastuzumab is widely used for targeted therapy of HER+ BC, although the therapy outcome varies between individuals [40]. While a patient is treated with trastuzumab, the drug action interferes with human peripheral blood endogenous factors. The most abundant HER ligand in human serum is EGF [42,43]. To check how human serum affects cell proliferation, we measured the impacts of individual human peripheral blood serum samples taken from 33 HER2+ BC patients and 17 healthy donors. We showed that cell sensitivity to trastuzumab greatly varies (up to 14 times for BC patients’ samples) among individual samples. Human serum samples act with trastuzumab in a synergistic, additive and antagonistic manner. This could explain the individual variation in the outcome of trastuzumab therapy between patients with HER2+ BC. In vitro, more reliable data can be obtained when drug activity is tested in the presence of human sera. Elsewhere inhibitory effect of the drug can be overestimated. The variability of serum effects on cell survival upon trastuzumab treatment opens an avenue for the development of new molecular diagnostics for personalized prediction of HER-targeted drug sensitivity. The identification of human serum factors that influence trastuzumab impact could specify how HER2-overexpressing cells respond to HER-targeted drugs treatment. We, therefore, assessed whether clonogenicity was associated with the serum concentrations of HER ligands and found statistically significant, yet relatively low, negative correlation of TGFa concentration and clonogenicity in the absence of trastuzumab but not when trastuzumab was added to the media. No other concentrations of HER ligands were associated with clonogenicity. Thus, further studies are needed to identify the human serum factors altering cell clonogenicity and sensitivity to trastuzumab.

Interestingly, in the absence of trastuzumab, human peripheral blood serum samples also inhibited BT474 clonogenicity (the mean was ~50% for the growth medium with 2.5% human serum). Moreover, we showed that cell growth varied up to ten times depending on which individual serum sample was used.

We showed that EGF suppresses trastuzumab activity for HER2-overexpressing BT474 cell growth. BT474 cell culture cannot serve as a comprehensive model for HER2+ BC, and similar studies with additional cell lines may be needed for an in-depth exploration of the inhibitory interplay of human blood serum and HER-targeted drugs. Similar result was obtained earlier for several HER-positive cell lines for interference between NRG1 and lapatinib, as well as between EGF and lapatinib and between EGF and cetuximab [36,37,38,39].

We showed that human sera differently influence BT474 cell growth. To study this, we measured cell-cycle distribution change upon cell treatment with different human serum samples. Interestingly, human serum significantly shifted down the G0/G1 phase and shifted up the G2/M phase, almost to the same extent; this shift level varied among individual samples. G2/M arrest can be responsible for cell growth inhibition [62].

Among the eight genes whose expression increased 4-fold and more in the presence of human serum, three genes were related to cell adhesion (CDH5, CLDN1, AZGP1). There were also two transcription factor genes: ZBTB16, involved in cell-cycle progression, and EYA2, involved in DNA double-strand break repair. This finding can at least partly explain the increased content of cells in the G2/M phase and decreased clonogenicity caused by human serum. 

When cells were treated with trastuzumab, the proportion of cells in the G0/G1 phase increased in agreement with cell growth inhibition results; similar results were obtained previously [61]. Trastuzumab inhibited cell growth to a lesser extent when acting with EGF; however, we found that G0/G1 was not shifted back to the control level. Thus, for the cell growth rate, EGF interference with trastuzumab cannot be explained by its influence on cell-cycle phase distribution.

To further investigate the mechanism of trastuzumab action on BT474 cells, we focused on genes that were significantly regulated by trastuzumab in the presence of FBS only or in the presence of donor serum but not altered in the presence of EGF (Table 2, Figure 8D). We hypothesized that these genes may be important for EGF-mediated rescue of BT474 cells from trastuzumab growth inhibition. Among these genes, *CYP1A2* (induced) can be mentioned, as it encodes a member of the cytochrome P450 enzyme superfamily and is thought to influence BC [41,63]. Another gene is *ODC1* (induced) for ornithine decarboxylase, which catalyzes the biosynthesis of precursors for polyamines spermine and spermidine, which are essential for cell proliferation and other cellular processes [64]. *CALCOCO1* (induced) encodes the N-terminal SKIP carboxyl homology domain, which is targeted to the endoplasmic reticulum. *CALCOCO1* levels are depressed in colorectal cancer; it may also function as a tumor suppressor and is related to cancer cell motility and metastasis [65]. The *CNKSR2* gene (induced) encodes the connector enhancer of kinase suppressor of Ras. *CNKSR2* product may function as an adapter protein or regulator of Ras signaling pathways [66]. Finally, the *CLEC7A* gene (induced) product activates the phosphorylation of SCIMP, an adapter protein involved in MHC class II signal transduction and immune synapse formation.

*PGM5* is an independent risk factor for overall survival. *PGM5* overexpression significantly inhibited the proliferation, invasion and migration abilities of CRC cells. On the contrary, a knockdown of *PGM5* promotes the invasion and migration of CRC cells. We found that *PGM5* was induced 7.8-fold by trastuzumab in the presence of human serum. However, no significant change of PGM5 expression was detected when trastuzumab treatment was applied without human serum [67]. In turn, EGF decreased trastuzumab induction 2.7-fold along with a decrease in trastuzumab inhibitory effect. *C8orf4* (inhibited by trastuzumab) is highly expressed in several tumors and is implicated in tumorigenesis; *C8orf4* augments Wnt/β-catenin signaling in cancer cells, suggesting that it may be involved in the regulation of self-renewal [68].

Moving to the level of molecular pathways, we showed that “Ribosome biogenesis in eukaryotes pathway” was downregulated by trastuzumab in the presence of donor serum. Deregulation of pathways controlling ribosome biogenesis has been observed in high-grade serous ovarian cancer; targeting ribosome biogenesis is promising for prostate cancers and colorectal cancer treatment [69].

We also found that the “Meiotic recombination pathway” was downregulated by trastuzumab. BRCA1 is a member of this pathway, which plays a crucial role in BC [70].

Another downregulated pathway was “PLK1 signaling events”. It was previously shown that inhibition of this pathway could lead to death of cancer cells by interfering with multiple stages of mitosis [71]. PLK1 is a key cell-cycle regulator, as well as an activator of MAPK signaling [72]; activation of the PLK1 pathway has been found in a variety of human cancers [71].

We found that the “Regulation of retinoblastoma protein (Rb)” pathway was downregulated by trastuzumab vs. FBS only. Rb binds and represses E2F transcription factors, which drive the expression of the S phase genes, thereby arresting cell-cycle progression [73]. E2F transcription factor family allows the control of the main Rb functions, while the loss of these interactions greatly enhances cancer development [74].

In total, we detected four cell signaling pathways downregulated by trastuzumab and no upregulated pathways. No pathways were significantly differential after trastuzumab treatment in the presence of EGF. Thus, the undesirable interference of EGF with trastuzumab inhibitory effect on BT474 cells can be explained by EGF action on the above-mentioned pathways.

GRN analysis revealed drastically different transcription factor networks required for the transition from control to trastuzumab-treated state with and without EGF. The FOXO1 transcription factor was the most crucial in the absence of EGF. It acts as a tumor suppressor and is a central regulator of cellular homeostasis [75]. In the case of EGF presence, the ETV1-ETV5 transcription factors were responsible for transcriptional changes induced by trastuzumab. This is in line with previous observation that ETV1 and ETV5 expression is enhanced by EGF stimulation [76].

We found that only one drug induced gene expression changes opposite to the ones caused by the trastuzumab treatment. This drug (SB-216763) inhibited Glycogen synthase kinase-3 beta (GSK-3), which is known to be a therapeutic target for numerous human diseases, including cancer. At the same time, GSK-3 might act as a tumor suppressor [77]. We showed that the same drug (SB-216763) caused gene expression changes similar to the ones caused by the trastuzumab and EGF treatment compared with EGF only. However, the trastuzumab inhibitory effect on BT474 cell proliferation cannot be attributed to GSK-3 repression only because the trastuzumab and EGF treatment does not inhibit cell growth to the same extent as trastuzumab only.

We found five compounds which caused gene expression changes similar to those caused by the trastuzumab treatment. All compounds were inhibitors of the PI3K/Akt/mTOR pathway [12,78]. The PI3K/Akt/mTOR pathway has been implicated in trastuzumab resistance in HER2+ BC; preclinical studies indicated that inhibitors of this pathway can act synergistically with trastuzumab in resistant cells [79]. However, EGF does not eliminate the transcriptional changes induced by trastuzumab, pointing at PI3K/Akt/mTOR inhibition. Thus, the CMAP analysis of the closest perturbagens does not decipher the mechanism of EGF action on trastuzumab-treated cells. Interestingly, the PKC-inhibitor had the effect opposite to trastuzumab and EGF but not trastuzumab without EGF. Previously, Fan et al. showed that EGFR signals to mTOR through PKC independently of Akt in glioma [80]. Thus, the addition of EGF may activate the PKC and thus rescue BT474 from the trastuzumab inhibitory effect. At the same time, a comparison between trastuzumab and human serum vs. human serum did not reveal any perturbagen whose action was similar to trastuzumab action in the presence of human serum. In order to investigate the mechanism of EGF action on trastuzumab-treated cells, it could be insightful to study whether the PI3K/Akt/mTOR signaling axis inhibitors decrease BT474 proliferation and investigate the possible modulation of their activities by human serum and EGF.

## Figures and Tables

**Table 1:** Alteration of cell-cycle distribution upon the treatment * with trastuzumab, EGF and human blood serum of BT474 cells compared with non-treated cells, in % **.

	G0/G1	*p*-Value	S	*p*-Value	G2/M	*p*-Value
EGF	+1.72	0.090	+0.3	0.800	−2.10	0.250
Trastuzumab	+5.91	<0.001	−2.91	<0.001	−3.00	0.090
Trastuzumab EGF	+4.11	<0.001	−2.42	0.050	−1.69	0.240
sm1	−4.37	0.004	+0.3	0.803	+4.02	<0.001
sd2	−6.67	<0.001	+2.9	0.064	+3.72	<0.001
sd3	−6.49	<0.001	+2.2	0.147	+4.24	<0.001
sd12	−7.42	<0.001	+2.7	0.089	+4.72	<0.001
sd13	−4.03	0.007	−0.62	0.689	+4.65	<0.001
sd14	−2.45	0.099	−0.83	0.582	+3.28	0.002
sd15	−2.04	0.177	+1.2	0.424	+0.80	0.057
human sera, mean	−4.44	<0.001	+1.3	0.395	+3.14	<0.001

* Trastuzumab concentration was 10 µg/mL; EGF concentration was 5 ng/mL, 5% human sera. ** In the non-treated BT474, the proportion of G0/G1 cells was 66.88 ± 4.10; in the S phase, the proportion was 9.48 ± 1.50; in the G2/M phase, the proportion was 23.64 ± 2.64 (%).

**Table 2:** Differentially expressed genes upon trastuzumab treatment of BT474 cells in the presence and in the absence of human serum, whose expression was not significantly changed in the comparison of trastuzumab vs. trastuzumab EGF treatment.

Gene ID	log_2_ (Fold Change)	
	Trastuzumab + FBS vs. FBS	Trastuzumab + Human Serum vs. Human Serum
*MEGF10*	1.94	2.61
*CACNA1C*	1.74	1.94
*CALCOCO1*	1.56	1.55
*B4GALNT3*	1.52	1.33
*TGFB2*	1.49	2.02
*BPIFB1*	1.45	1.33
*HMGCS2*	1.44	1.60
*CYP1A2*	1.33	2.90
*PSG4*	1.29	2.74
*ITGA9-AS1*	1.28	1.05
*MYO15B*	1.24	1.50
*PTPRN2*	1.20	1.30
*TCP11L2*	1.19	1.31
*REG4*	1.18	1.42
*PEX11G*	1.18	1.01
*LDLRAD1*	1.15	1.35
*YPEL2*	1.12	1.17
*YPEL3*	1.09	1.10
*LINC02233*	1.08	1.46
*PDE11A*	1.07	1.21
*NATD1*	1.04	1.14
*TNFRSF11B*	1.02	1.20
*OSER1-AS1*	1.01	1.14
*ODC1*	−1.09	−1.24
*CNKSR2*	−1.17	−1.17
*ROBO2*	−1.18	−1.35
*PTH2R*	−1.27	−1.56
*GNAZ*	−1.41	−1.04
*ODAM*	−1.43	−2.24
*CD101*	−1.51	−1.11
*RCOR2*	−1.52	−1.22
*CLEC7A*	−1.60	−2.71
*GAL*	−2.06	−2.51

## Data Availability

Not applicable.

## Supplementary Materials

The following supporting information can be downloaded at: https://www.mdpi.com/article/10.3390/biomedicines10081914/s1, Figure S1: Colony formation by BT474 cells in the growth media containing 7.5–17.5% of heat-inactivated FBS; Table S1: Age of blood donors; Table S2: Colony formation of BT474 cells in the presence of trastuzumab and 2.5% healthy human serum (samples sd) or HER2-positive BC patients (sp).
