# Supplementary material for: Human Blood Serum Inhibits Ductal Carcinoma Cells BT474 Growth and Modulates Effect of HER2 Inhibition
Source: Biomedicines. 2022 Aug 8;10(8):1914. doi: 10.3390/biomedicines10081914 (PMC9405390; doi:10.3390/biomedicines10081914)
Supplement: Supplementary file 1 [file biomedicines-10-01914-s001.zip › supplementary tables 1-2 and figure 1.pdf]

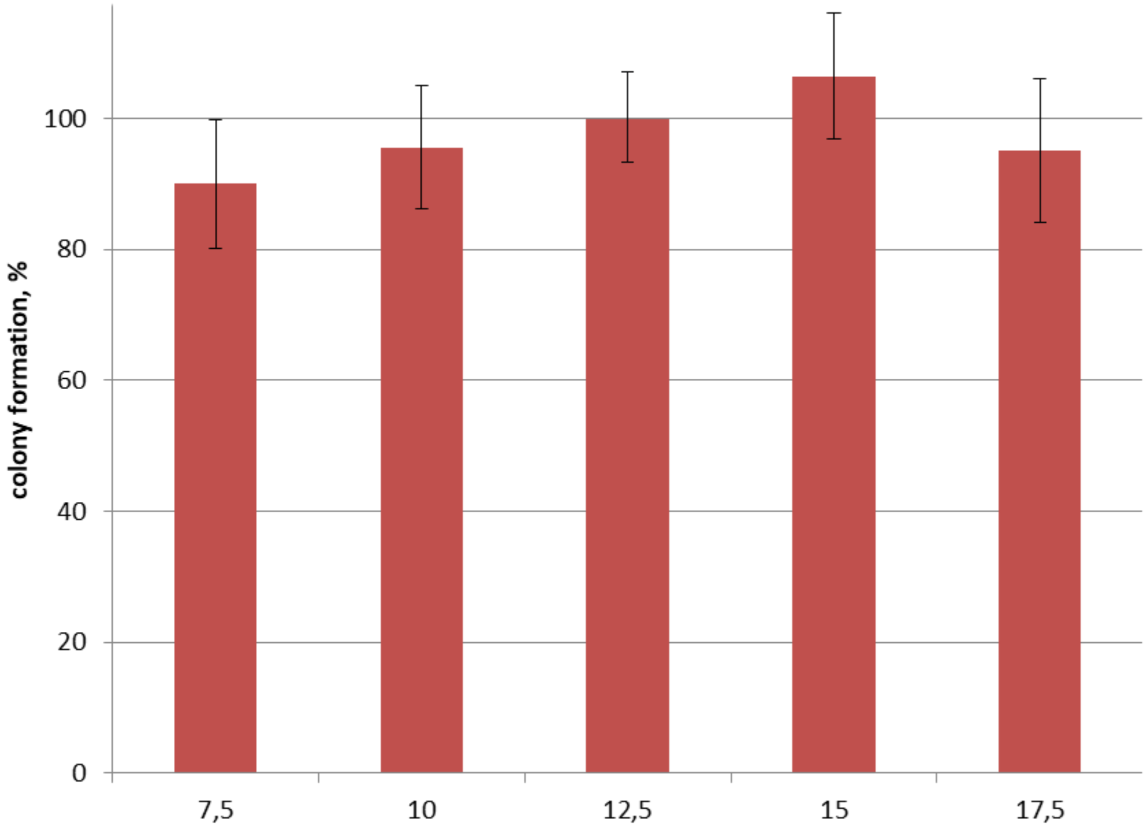

Figure S1. Colony formation by BT474 cells in the growth media containing with 7.5 – 17.5% of heat-inactivated FBS. Colony formation is normalized to the growth media containing 12.5% FBS. The data is presented as mean ± standard deviation from three independent replicates.

Table S1. Age of blood donors.

Healthy donors	age, years		BC patients	age, years
Sd1	40		sp1	68
Sd2	21		sp2	69
Sd3	45		sp3	63
Sd4	64		sp4	48
Sd5	38		sp5	72
Sd6	54		sp7	41
Sd7	35		sp8	36
Sd8	59		sp9	55
Sd9	30		sp10	43

Sd10	38		sp11	49
Sm 1	53		sp12	53
sd12	53		sp13	52
sd13	32		sp14	37
sd13	33		sp15	36
sd14	21		sp16	32
sd15	32		sp17	62
sd16	33		sp18	31
			sp19	50
			sp20	50
			sp21	61
			sp22	55
			sp23	38
			sp24	60
			sp25	70
			sp26	60
			sp27	61
			sp28	72
			sp29	72
			sp30	62
			sp31	52
			sp32	63
			sp34	56
			sp35	53

Table S2. Colony formation of BT474 cells in the presence of trastuzumab and 2.5% healthy human serum (samples sd) or HER2 positive BC patients (sp). The number of colonies in each experiment is normalized by number of colonies in the absence of the drug and human serum. The data are given as mean ± standard deviation.

Serum sample	no drug	Trastuzumab, 0.5 µg/ml	Bliss Synergy Score
FBS	100 ± 9.7	51 ± 3.2	
Sm1	23.3 ± 3.2	13.4 ± 3.2	-1.52141
Sd1	53.26 ± 7.2	19.15 ± 3.2	8.017635
Sd2	44.66 ± 5.7	31.05 ± 3.7	-8.28068
Sd3	59.46 ± 4.8	9.95 ± 2.8	20.3762
Sd4	39.65 ± 5.5	8.7 ± 3.5	11.52498

Sd5	42.47 ± 6.3	15.68 ± 3.3	5.980582
Sd6	49.14 ± 6.2	12.81 ± 3.2	12.246
Sd7	38.58 ± 5.8	15.04 ± 3.8	4.639139
Sd8	42.65 ± 6.7	53.8 ± 3.7	-32.0456
Sd9	78.34 ± 7.3	13.25 ± 3.3	26.70103
Sd10	70.76 ± 7.3	14 ± 3.3	22.08804
Sd12	60.21 ± 6.8	9.2 ± 2.8	21.50933
Sd13	62.2 ± 7.6	11.76 ± 3.6	19.96289
Sd14	35.41 ± 3.2	13.27 ± 3.2	4.795251
Sd15	88.07 ± 7.3	20.65 ± 3.3	24.26283
Sd16	60.82 ± 7.1	17.7 ± 3.4	13.31647
sd13b	65.65 ± 6.8	38.94 ± 3.4	-5.46211
Sp1	46.99 ± 6.1	10.92 ± 3.4	13.04111
Sp2	67.67 ± 7.4	15.74 ± 3.2	18.7749
Sp3	60.37 ± 7.9	11.85 ± 3.3	18.9401
Sp4	49.27 ± 6.4	13.51 ± 3.6	11.61259
Sp5	42.73 ± 6.7	15 ± 3.1	6.796779
Sp 7	81.51 ± 9	23.14 ± 4.5	18.427
Sp 8	76.34 ± 7.6	31.84 ± 3.7	7.089096
Sp 9	38.02 ± 6.5	17.59 ± 3.7	1.800825
Sp 10	71.62 ± 7.6	23.7 ± 4.6	12.83068
Sp 11	49.57 ± 6	20.73 ± 4.7	4.547621
Sp 12	72.53 ± 7.4	18.14 ± 3.8	18.8499
Sp 13	51.7 ± 6.1	15.18 ± 3.7	11.18726
Sp 14	72.69 ± 7.3	17.64 ± 3.1	19.43229
sp15	67.69 ± 7.1	13.87 ± 3.7	20.65468
sp16	56.29 ± 6.9	9.05 ± 3.2	19.66397
sp17	38.76 ± 6	11.76 ± 3.2	8.011188
sp18	77.53 ± 7.4	16.81 ± 3.2	22.73081
sp19	68.91 ± 7.3	3.69 ± 3.2	31.45288
sp20	64.61 ± 7.9	8.44 ± 3	24.50757
sp21	61 ± 7.2	13.28 ± 2.1	17.83336
sp22	55 ± 6.8	5.75 ± 1.4	22.29679

sp23	26.49 ± 4.7	6.2 ± 2.2	7.315429
sp24	24.25 ± 3.3	4.43 ± 1.3	7.943856
sp25	52.24 ± 4.3	7.97 ± 2.6	18.67581
sp26	28.36 ± 5.8	10.62 ± 3.1	3.841375
sp27	60.02 ± 6.6	26.55 ± 3.5	4.055292
sp28	35.54 ± 3.2	15.05 ± 2.7	3.078314
sp29	8.21 ± 2.3	1.77 ± 1.7	2.416348
sp30	45.77 ± 3.2	20.8 ± 2.6	2.540985
sp31	58.07 ± 5.1	10.62 ± 1.7	18.99291
sp32	53.74 ± 5.4	5.31 ± 1.8	22.09902
sp34	28.73 ± 3.3	17.7 ± 2.7	-3.0492
sp35	66 ± 5.2	17.7 ± 2.1	15.95876
